# Supplementary material for: MetaRibo-Seq measures translation in microbiomes
Source: Nat Commun. 2020 Jun 29;11:3268. doi: 10.1038/s41467-020-17081-z (PMC7324362; doi:10.1038/s41467-020-17081-z)
Supplement: Supplementary file 10 — Supplementary Data 7 [file 41467_2020_17081_MOESM10_ESM.zip › File2/Confidence_High_Taxonomy/191084_out.krona.html]

Javascript must be enabled to view this page.

members
magnitude
magnitudeUnassigned
count
unassigned
taxon
rank

191084\_out

2

2
superkingdom
2

1239
2
phylum

186801
2
class

186802
2
order

186803
family
2

genus
1
572511

40520

SRS146813\_contig\_number\_contig-100\_22058.22058
1
species

species
1

SRS077641\_contig\_number\_15129
39491
